# Inhibition of ammonia and hydrogen sulphide as faecal sludge odour control in dry sanitation toilet facilities using plant waste materials

**DOI:** 10.1038/s41598-021-97016-w

**Published:** 2021-09-07

**Authors:** Bernice Mawumenyo Senanu, Patrick Boakye, Sampson Oduro-Kwarteng, Divine Damertey Sewu, Esi Awuah, Peter Appiah Obeng, Kobina Afful

**Affiliations:** 1 Department of Civil Engineering, Kwame Nkrumah University of Science and Technology, PMB, UPO, Kumasi, Ghana; 2 Department of Chemical Engineering, Kwame Nkrumah University of Science and Technology, PMB, UPO, Kumasi, Ghana; 3 Life Green Technology Co. Ltd., 875 Yuseong-daero, Yuseong-gu, Daejeon, 34158 Republic of Korea; 4 Department of Chemical and Biological Engineering, Hanbat National University, 125 Dongseo-daero, Yuseong-gu, Daejeon, 34158 Republic of Korea; 5 Department of Water and Sanitation, University of Cape Coast, Cape Coast, Ghana

**Keywords:** Environmental sciences, Diseases, Engineering

## Abstract

On-site dry sanitation facilities, although cheaper than wet sanitation systems, suffer from high malodour and insect nuisance as well as poor aesthetics. The high odour deters users from utilizing dry sanitation toilets as an improved facility leading to over 20% open defecation in Sub-Saharan Africa. To address this malodour concern, this study first assessed odour levels, using hydrogen sulphide (H_2_S) and ammonia (NH_3_) as indicators, on two dry sanitation facilities named T1 and T2. The potential of using biomass (sawdust, rice husk, moringa leaves, neem seeds), ash (coconut husk, cocoa husk) or biochar (sawdust, rice husk, bamboo) as biocovers to remove or suppress odour from fresh faecal sludge (FS) over a 12-day period was investigated. Results showed that the odour levels for H_2_S in both T1 (3.17 ppm) and T2 (0.22 ppm) were above the threshold limit of 0.05 ppm, for unpleasantness in humans and vice versa for NH_3_ odour levels (T1 = 6.88 ppm; T2 = 3.16 ppm; threshold limit = 30 ppm limit). The biomasses exhibited low pH (acidic = 5–7) whereas the biochars and ashes had higher pHs (basic = 8–13). Basic biocovers were more effective at H_2_S emission reduction (80.9% to 96.2%) than acidic biocovers. The effect of pH on suppression of NH_3_ was determined to be statistically insignificant at 95% confidence limit. In terms of H_2_S and NH_3_ removal, sawdust biochar was the most effective biocover with odour abatement values of 96.2% and 74.7%, respectively. The results suggest that biochar produced from locally available waste plant-based materials, like sawdust, can serve as a cost-effective and sustainable way to effectively combat odour-related issues associated with dry sanitation facilities to help stop open defecation.

## Introduction

Poor sanitation is a major cause of poverty and some preventable diseases like diarrhoea, intestinal worms and dysentery^1^. The lowest sanitation coverage is concentrated mainly in countries in Sub-Sahara Africa and Southern Asia^2^. Populations living in urban centres in many developing countries lack household toilets and the only toilet facility is the shared toilet systems^3^ meant for public use—markets, transport stations and schools. Ghana’s sanitation coverage, as of 2017–2018 was 21%; which is below the millennium development goal (MDG) target of 54%^4,5^. On-site sanitation technology in Ghana serves 85% of the population^6^, of which 68.2% use public toilets and 19.3% practice open defecation^7^. For dry sanitation toilets, 29% of the population use pit or ventilated improved pit (VIP)^7^ whereas users of water closets account for 15.4% of the population. Dry on-site sanitation technologies are relatively cheaper, require little or no water and occupy relatively less land, but the facility is usually characterized by malodour and insect nuisance^8^, which can discourage users of the facility from patronizing it and rather resort to open defecation^9,10^.

Malodours are normally indicators that protect humans from potential illness caused by infection through contaminated food and matter^11^. The odours are generally attributed to the evolution of different smell-causing substances (volatile compounds) arising from the anaerobic decomposition of the faecal matter^12–14^. The type of volatile compound evolved is also dependent on the age of faecal matter where fresh ones have rancid odour whereas aged ones in latrines have sewage, malodorous smell, like rotten egg due to the anaerobic decomposition process^13^. The rancid and cheesy odour in dry latrines is associated with the evolution of volatile compounds such as phenylacetic acids, butyric, isovaleric, 2-methyl butyric, isobutyric valeric and hexanoic. Sewage, rotten egg and rotten vegetable odours have been attributed to sulphur-based volatile compounds—arising from protein degradation and activities of sulphur-reducing bacteria^15,16^—such as dimethyl trisulphide, hydrogen sulphide (H_2_S), dimethyl disulphide, methyl mercaptan and dimethyl sulphide. Also, skatole, p-cresol, some carboxylic acids, phenol and indole have been associated with farmyard manure-like odours^13,17–19^. That notwithstanding, the sulphur and nitrogen-containing compounds, particularly ammonia (NH_3_) and H_2_S, are of particular importance since they are the primary odorous substances and possess a distinctive odour that is readily noticeable even in small concentrations [H_2_S = 0.005 ppm^20^; NH_3_ = 0.05 ppm^21^]^22^. In fact, a positive correlation between H_2_S concentration and user perception of odour have been recorded; otherwise for NH_3_ concentration^8^. It is, therefore, no wonder that recommendations about the odour-irritation threshold concentrations of the NH_3_ and H_2_S have been enacted and thus, respectively, ranges from 4 to 8 ppm and from 2.5 to 20 ppm^23^. Also, to avoid complaints from the facility users, it is recommended that the concentration of the H_2_S should not exceed 7 µg/m^3^ (0.05 ppm) for a 30-min averaging period^24^.

Many approaches such as pH alteration, specialized/engineered microorganisms usage, microbial growth inhibition and use of biological covers (biocovers) have been investigated to address the malodorous nuances associated with the usage of dry sanitation toilets^25,26^. Biocovers, in particular, are materials that serve as covers over faecal matter to help suppress gas emissions by either physically limiting the emissions of gases from the surface of the faecal matter or creating a biologically active zone on the top of the biocovers where gases are aerobically decomposed by microorganisms^20^. Biocovers may be impermeable or permeable to gases depending on the material used. Impermeable biocovers only trap the odorous substances and are therefore normally used in conjunction with other treatment methods such as biofilters or scrubbers^26^. Examples include glued layers of polyethylene film and tarpaulin^27^. Permeable biocovers, however, act like biofilters and can trap and subsequently biotransform odorous gases to harmless or less odorous forms^26^. For instance, H_2_S evolution can be inhibited via components in the biocovers reacting with and converting the dissolved sulphide into other intermediate forms, or inert metallic sulphides, or bisulphide ions^20^. The performance of the biocovers is, therefore, dependent on their physicochemical properties—surface area, porosity, mineral composition, organic matter content and pH amongst many others^28^—and thickness of the applied biocover layer^20^. It is known that NH_3_ evolution can be attenuated in low pH^26^. High organic matter, surface area, porosity and cation exchange capacities (CEC) of waste biocover soil were effective at mitigating H_2_S evolution via adsorption, principally^28^. It is, therefore, no wonder that permeable biocovers with the aforementioned properties have been investigated. These include waste lignocellulosic agricultural biomass—mulched wood material^29^, cornstalks, straws and wood chip^30^—, geotextile fabrics^31^, polystyrene foams^32^, silicates or clays^33^, fly ash^29^ and combinations of zeolite and agricultural biomass^34^. Lignocellulosic agricultural biomass, for instance, generally contains high organic matter content, helpful as food for microorganisms. Ash is known to contain inorganic constituents especially of alkali and alkaline-earth metals, which renders it highly basic^35^ and as such can abate H_2_S release via its adsorption capability and potential acid–base reactions with acidic H_2_S^36^ when used as a biocover. Another potential biocover seldom researched is biochar.

Biochar, the carbonaceous product of biomass pyrolysis, has gained much popularity as a promising material for different high-value applications such as waste management and climate change mitigation tool^37,38^. Biomass for biochar production can be sourced from locally available agricultural wastes, making it cheap and conducive to the environment^39–41^. Many studies have shown biochar to be an effective remediation tool for diverse pollutants of organic and inorganic origin^42^. For instance, heavy metals such as Pb^43^ and Cd^44^, and radioactive isotopes like Sr^45^ and Ce^46^ have been successfully removed with biochar. Furthermore, other pollutants like dyes^47^, pesticides^48^, pharmaceuticals^49^, and NH_4_-N, N_2_O and P^50^ have also been successfully immobilized, mineralised and/or trapped within biochar. The removal of these pollutants have mostly been ascribed to the unique physicochemical properties of the biochars such as large specific surface area, high porosity, moderate CEC, abundant surface functionality, inorganic contents, and excellent thermal, mechanical and chemical stability^51,52^; which may come in handy for odorous compounds mitigation. It is, therefore, hypothesized that given the aforementioned unique physical and chemical characteristics of biochars, they may serve as potentially excellent biocover to mitigate odour release from dry sanitation toilets.

This study, therefore, investigates the application of biomass, ash and biochar as potential biocovers to attenuate odour evolution from fresh faecal sludge (FS) generated in dry sanitation toilets. The specific objectives of this study are to (1) determine the on-site odour levels of dry-sanitation public toilets using NH_3_ and H_2_S, as the primary odour-indicators; (2) acquire, produce and characterize different materials as potential biocovers for odour mitigation; and (3) evaluate the odour-suppression or odour-removal efficiencies of the as-produced biocovers on fresh human excreta samples from the dry sanitation toilets in a laboratory setting.

## Materials and methods

### Study setting and description of the VIP latrines

Faecal samples for this study were obtained from public toilets in Ayeduase. Ayeduase is a community located in the Oforikrom Sub-Metro of Kumasi Metropolitan Assembly in the Ashanti Region in Ghana with a human population estimated at 29,748 and has 6° 40′ 0′′ N and 1° 34′ 0′′ W in DMS (Degree Minutes Seconds) as its coordinates^53^. Apart from student hostels and other well-built houses with wet toilet facilities, the majority of the natives use dry on-site toilet systems including shared facilities. This is because many of the houses are old structures built without toilet facilities. So inhabitants are compelled to use the nearest available public toilets. Some dwellers share public toilets intended for public schools.

For this research, two public, dry on-site toilets (VIP latrines) were considered. The first public toilet (TI), which is located close to the Ayeduase market has depth beyond 2.5 m and houses ten (10) squat holes; one-half dedicated to each sex with an inter-squat hole concrete-partition-separation. An average of seventy-five (75) people use the toilet daily with a quarterly de-sludging frequency every year. The second public toilet (T2), is located close to the Ayeduase school, has a depth of almost 2 m and equipped with twelve (12) squat holes—two sets of five squat holes placed back to back on opposite sides of a dividing wall, with one set assigned to males and the other set to females. Every squat hole within a set is separated from each other by a concrete partition. The remaining two squat holes were adjacent to the five squat holes and contained in enclosed rooms. Over ninety (90) residents and school children patronize this toilet facility and are de-sludged every other week. Both TI and T2 were fitted with vent pipes at heights exceeding 500 mm above the roof of the superstructure.

### Determination of on-site odours from VIP latrines

To determine the degree to which odour was a nuisance in the use of VIP latrines, direct on-site measurements of H_2_S and NH_3_ concentrations, representing odour, were carried out in the enclosures of T1 and T2 without the need for gas collection. Odour readings were taken from both T1 and T2, consistently for ten (10) days; three times daily: morning (6:30–7:30), afternoon (12:30–13:30) and evening (17:30–18:30) in greenwish mean time (GMT). The odour-causing gases were detected and quantified via direct air measurements in the enclosures of T1 and T2 using an aeroqual potable gas analyser (series 200, New Zealand).

### Sampling protocol of FS for laboratory-based experiments

FS from both T1 and T2 was sampled from the surface of the pile of excreta beneath the pit pedestal. At the time of sampling, FS level in TI and T2 was, respectively, about 2 m and 10 cm, away from the squat hole. The sampling was undertaken by scraping off the top of the excreta with a one-meter-long ladle-like tool, to obtain a representative “fresh” faecal matter sample. The samples were collected into a tightly capped, 2000 ml plastic bucket and transported to an environmental laboratory in the civil engineering department of Kwame Nkrumah University of Science and Technology (KNUST) for analysis.

### Acquisition and production of biocovers for odour mitigation

**Figure 1:**
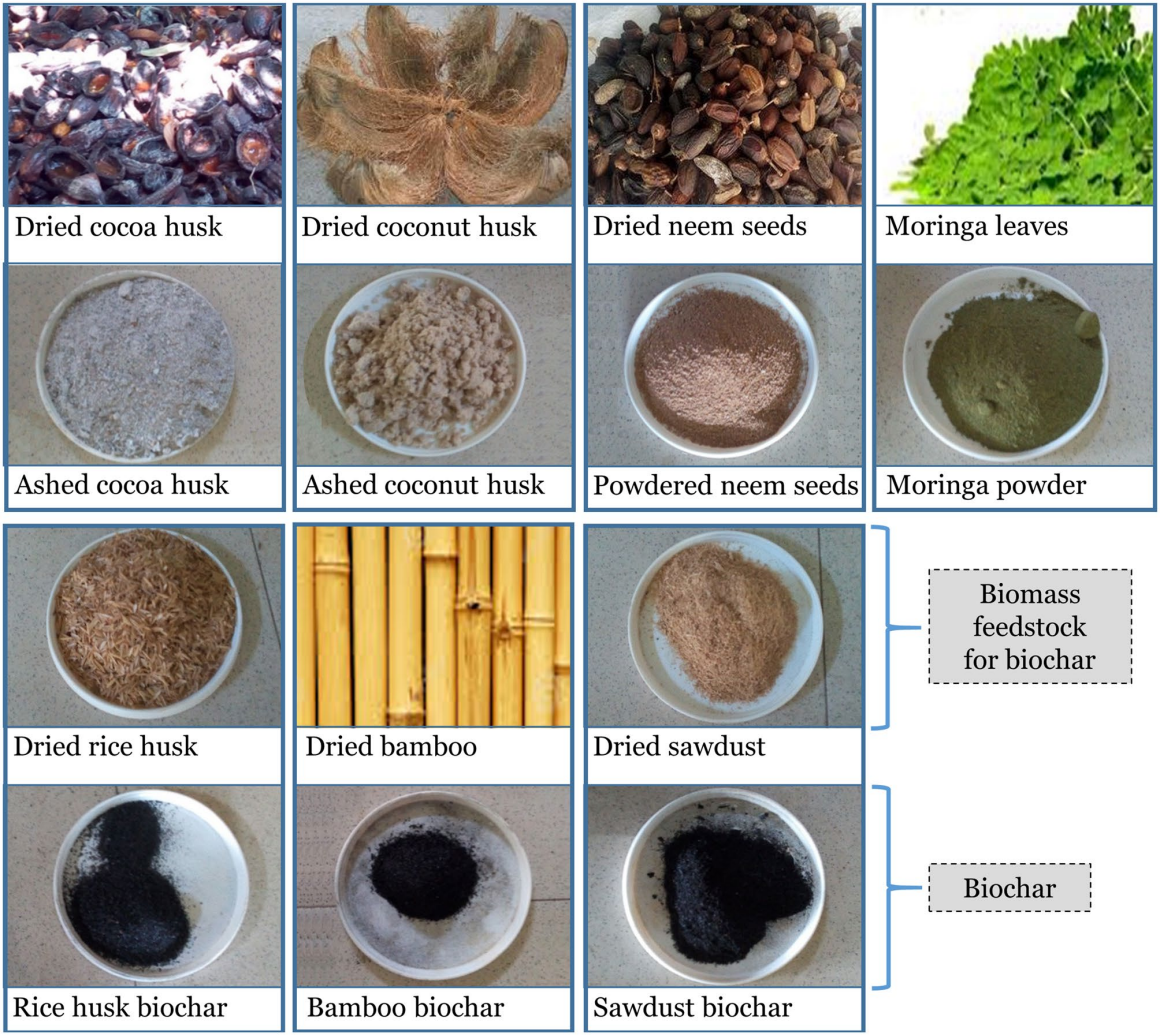
Photographs of the various waste biomasses and their corresponding ashes or biochars employed as biocovers in this study.

Locally available materials including agricultural waste were used as potential biocovers for the mitigation of odour release from FS. Seven (7) biomasses were obtained for this experiment: sawdust (SD) from *Celtis Mildbraedii*, rice husk (RH), moringa leaves (M), neem seeds (NS), cocoa husk (CH), coconut husk (C_N_H) and bamboo (B) (See Fig. 1). Some of these biomasses were used, as is—sawdust, rice husk, moringa leaves, neem seeds—or were thermally treated via pyrolysis and ashing operations.

For pyrolysis, only biomasses from rice husk, bamboo and sawdust were utilized as feedstock. The desired biomass was weighed and fed into the pyrolysis reactor with pyrolysis conditions of 400 °C for 90 min of sustained pyrolysis. After pyrolysis, the produced biochar was left to cool in the reactor for about 30 min before transferring into tightly capped vials to be stored for further experiments.

For ash production, only biomasses from the cocoa husk and coconut husk were used. Each biomass was fed into a kiln (HT13T7, Kiln and Furnace Limited, Keele St. Tunstall, Stoke-On-Trant) where ashing was undertaken at a temperature of 700 °C. After ashing, the kiln was switched off and allowed to cool to within 60 °C and 80 °C. The ashes were collected and stored in tightly capped glass vials to be used for further experiments. Note that all acquired and produced biocovers were ground to within 210 to 75 µm and used for further experiments. Acronyms of B for biomasses, BC for biochars and A for ash were attached to the feedstocks for each process to help in identifying the thermal condition employed. Consequently, the obtained biocovers were tagged as SD-B, RH-B, M-B, and NS-B for biomasses of sawdust, rice husk, moringa powder and neem seeds powder, respectively. Tags were also assigned to sawdust biochar (SD-BC), rice husk biochar (RH-BC) and bamboo biochar (B-BC). Biocovers from the ash were also tagged for cocoa husk ash (CH-A) and coconut husk ash (C_N_H-A).

### Evaluating the effect of additive application as biocovers for malodour mitigation

**Figure 2:**
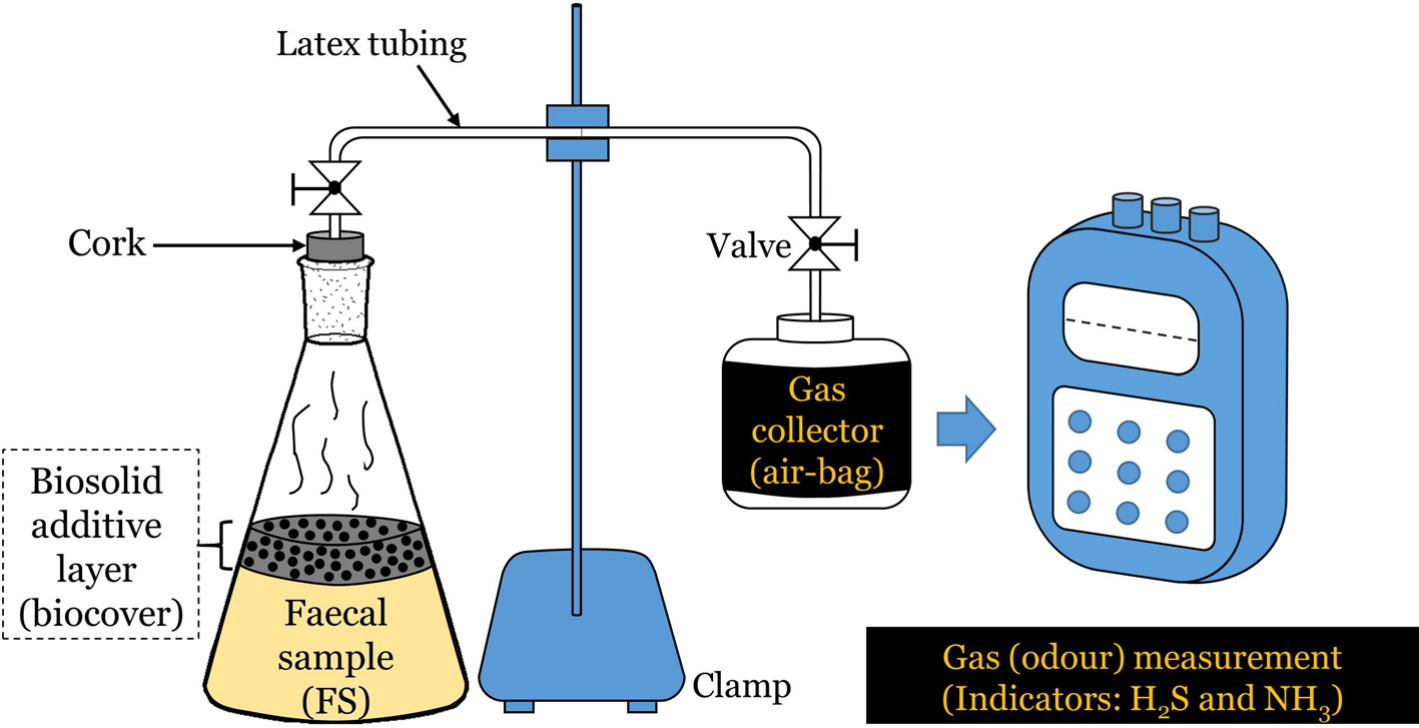
Experimental setup depicting additive application as biocovers for malodour mitigation.

The experimental setup is shown in Fig. 2. The experimental design is a completely randomized design with two replicate measurements. The desired mass of each additive—biomass from sawdust, powdered rice husk, powdered moringa leaves and neem cake; ash from the cocoa husk and coconut husk and; biochar from sawdust, rice husk and bamboo—corresponding to 5wt.% (1:20 w/w) was determined and transferred into a 500 ml conical flask containing 300 g of fresh FS. Care was taken to ensure complete coverage of the FS in the conical flask with the applied biocovers. A control sample, which contained only FS without additives, was also include to facilitate the determination of the odour-removal efficiencies of the biosolid additives. Each conical flask was fitted with a single-perforated tightly fitting cork connected with a latex tubing that ends in 500 ml air-bag for gas trapping for further analysis. Analysis of the trapped gases was undertaken every three (3) days for 12 days. To prevent leakages, all openings around the corks were sealed with a sealant. The experiments were performed in two replicates. The performance of the biocover was evaluated based on the per cent reduction in odour—NH_3_ or H_2_S—using Eq. (1).

1 $$ \% Odour\;reduction = \left( {\frac{{C_{1} - C_{2} }}{{C_{1} }}} \right) \times 100 $$

where *C*_1_ is the odour of the control sample (faecal sample without biocover) at a gas sampling time; *C*_2_ is the odour of the biocover-applied faecal sample at that same gas sampling time.

### Characterization experiments and statistical analysis

The fresh FS samples were analysed for chemical oxygen demand (COD—dichromate approach)^54^ and biochemical oxygen demand (BOD) using the Winkler method. Both the FS and biosolid additives (biocovers) were analysed following the standard methods for water and wastewater analysis for the determination of total organic carbon (TOC), fixed solids (ash), total volatile solids (TVS) and total solids (TS)^54^. TKN content for FS and biosolid additives (biocovers) was determined following the EN 13342 standard^55^. Also, pH was determined using a calibrated pH meter (Palintest micro 800 Mult, Singapore). The trapped gases in the airbags from the experimental setup in “Evaluating the effect of additive application as biocovers for malodour mitigation” section were analysed qualitatively and quantitatively for H_2_S and NH_3_ gases—representative of the malodorous gases—using the biogas 5000 gas analyser. Also, a comprehensive statistical analysis [two-way and one-way analysis of variance (ANOVA)] using the data analysis add-in in Microsoft® Excel was performed. The effect of two independent variables (biocover type and duration of biocover application) on suppression of odour (response variables: H_2_S and NH_3_) were investigated using the two-way ANOVA without replication function. The one-way ANOVA was, however, employed to assess the statistical differences between the applied biocover types (biomass, biochar, ash) on the overall odour suppression for the entire duration of the experiment. A confidence level of 0.05 was chosen as the basis to either reject or fail to reject the null hypothesis of no statistically significant difference for the comparison. The Tukey–Kramer multiple comparison test was utilized for specificities in cases where the null hypothesis was rejected.

### Consent to participate

All authors confirm participatory consent.

### Consent for publication

All authors accept to publishing in this journal.

## Results and discussions

### On-site odour evolution

#### Variations in H_2_S concentration on public toilets

**Figure 3:**
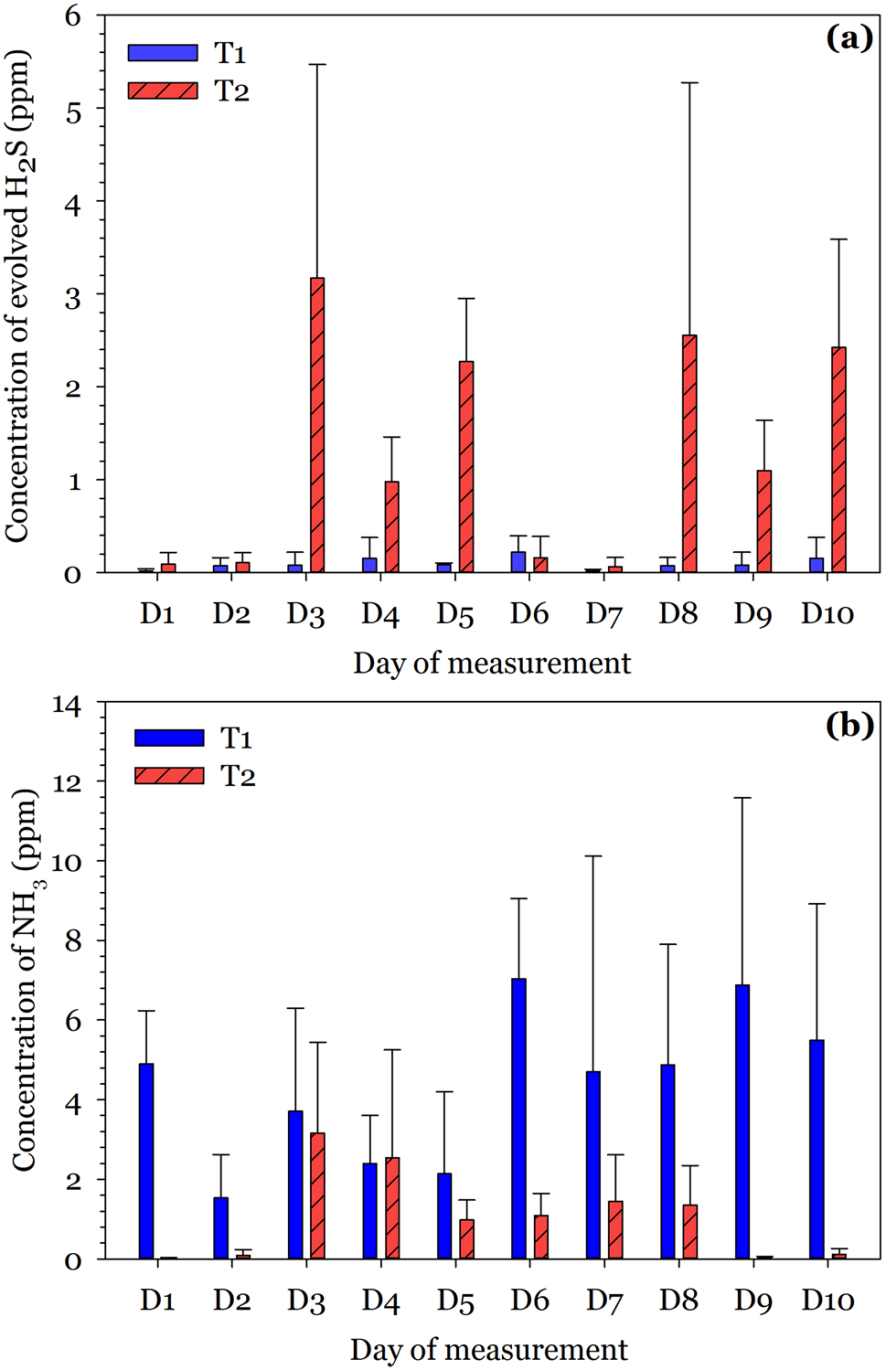
The concentration of H_2_S (**a**) and NH_3_ (**b**) released from T1 and T2 over 10 days as daily averages of the three sampling periods (mornings, afternoons and evenings).

The daily H_2_S concentrations collected over different times within the day for both T1 and T2 during the 10-day survey are available in the Supplementary Fig. S1a and the corresponding daily averages shown in Fig. 3a. Generally, the highest H_2_S concentration in the public toilet occurred during the mornings and evenings (Supplementary Fig. S1a); expectedly a consequence of the most patronized times in the day. Other factors such as user conduct and effectiveness of cleaning activities may have played a role. From Fig. 3a, it was evident that, except for day 6, daily averages of H_2_S was higher in T2 than T1, with some concentrations as high as 26.8 (day 5), 34.0 (day 8) and 39.2 (day 3) times that of T1. This could be attributed to the depth of the sludge in the pit of T1 (less than one-third of the pit depth) and T2 (almost filled to the brim), which is a consequence of the patronage frequency and cleaning activities. Consequently, more H_2_S will escape from the pit into the privy room in the case of T2 than T1, even though both facilities were fitted with vent pipes. Also, H_2_S is heavier (density of 1.36 kg/m^3^) than air (density of 1.225 kg/m^3^) and as such usually lingers at the base of the latrine^56^ even when fitted with vent pipes. Similar observations have been made by other authors who ascribed the observations to a large number of users^57^. Clearly odour in both T1 and T2 was detectable [> 0.005 ppm^20^]. Except for day 1 (0.021 ppm) and 7 (0.018 ppm) for T1, the daily H_2_S averages exceeded the guideline value of 0.05 ppm^8,24^ for all toilet facilities investigated, which will inevitably elicit complaints from users and residents, and hamper patronage of the toilet facilities.

#### Variations in NH_3_ concentration on public toilets

The concentration of NH_3_ was another component of odour that was measured in the two public latrines. Supplementary Fig. S1b shows the NH_3_ concentration detected at different times in the day, during the 10-day experiment in T1 and T2. There was no observable trend for NH_3_ evolution based on the sampling times. However, the daily averages (Fig. 3b) showed a higher evolution of NH_3_ for T1 than for T2 (except for day 4). Except for day 2, 3, 4 and 5, most of the daily NH_3_ released were within the detectable threshold of 4 to 8 ppm for humans for T1 (day 1 = 4.90 ppm; day 6 = 7.04 ppm; day 7 = 4.70 ppm; day 8 = 4.88 ppm; day 9 = 6.88 ppm; day 10 = 5.49 ppm). For T2, however, none of the readings went beyond the detectable threshold for humans. It is noteworthy that all measured NH_3_ concentrations were below the threshold of unbearableness and irritation for humans [(10 min exposure at 30 ppm—slight irritation; 10 min to 2 h exposure at 50 ppm—moderate irritation to the eyes, nose, throats and chest)^58^]. According to Strande and Brdjanovic^57^, factors such as diet, climate, type of toilet facility, number of users among others influence odour release. Differences in the NH_3_ measurement for T1 and T2 were attributed to the level of FS in the pit. Level of FS in TI and T2 were respectively, about 2 m and 10 cm away from the squat hole suggesting a poor maintenance regime especially for T2 since desludging should be undertaken when the sludge is about 50 cm from the slab. As such, NH_3_ which is less dense (0.73 kg/m^3^) than air (1.23 kg/m^3^) at 15 °C at sea level, is more likely to escape easily into the atmosphere for T2 since the FS, in this case, was much closer to the squat hole; this may explain why less NH_3_ was measured in T2 compared to T1. As such, the gas detector potentially recorded less NH_3_ concentration in the privy room. For T1 because the sludge depth in the pit was less than one-third the depth of the pit, enough room was available for NH_3_ to linger in the pit, which accounted for the reported higher NH_3_ concentrations.

Generally, it can be observed that NH_3_ concentrations recorded in both toilets were higher than that for H_2_S in the latrine (Fig. 3). The NH_3_ and H_2_S results in this study were in agreement with that obtained by Obeng et al.^8^, where mean NH_3_ concentration in ventilated improved pit public toilets was higher (2.99 ppm) than that for H_2_S (0.13 ppm).

### Characterization of FS

Table 1 shows the characteristics of FS. The quotient of COD to BOD (COD/BOD ratio) obtained in this study was two (2); which is low compared to others reported in the literature. For instance, FS with a COD/BOD ratio of 5 and 6 was reported by Strande and Brdjanovic^57^ and Jeuland et al.^59^ for public toilets, respectively. Strande and Brdjanovic^57^ intimated that the higher value of 5 was indicative of the slow degradation of organic matter. Besides, it is also known that the characteristics of FS vary depending on parameters such as diet, type of toilet technology, climate and the type of cleansing material utilised^8^. These can thus explain the variations of the COD/BOD ratio.

**Table 1:** Characteristics of the fresh faecal sludge.

Parameter	Unit	Mean	Standard deviation
Chemical oxygen demand	mg/l	181,900	± 17,678
Biochemical oxygen demand	mg/l	102,300	± 3818
Total Kjeldahl nitrogen	mg/l	15,500	± 0.03196
Moisture content	%	80.5	± 2.120
Total volatile solids	%	80.5	± 2.121
Total organic carbon	%	44.73	± 1.181
Ash	%	17.75	± 0.3536
pH		5.7	± 0.02121

Moisture content in FS obtained from the public toilet was high (80.5%) as expected. Strande and Brdjanovic^57^ reported a similar result of high moisture content (83%) for dry VIP latrine sludge. Moisture content values ranging from 53 to 92% have been reported^60^. The weather condition was identified as a contributing factor to the variations obtained. The pH of FS in this study was slightly acidic (pH 5.7) with a TKN of 15,500 mg/l (1.5%), which is particularly low. Nevertheless, according to Rodhe et al.^61^ although FS is usually rich in nitrogen, changes in the expected nitrogen content in FS is largely subject to the diet of the user. For example, a highly proteinaceous diet will result in higher nitrogen content in FS^57^.

### Characterization of biocovers employed as odour-reducing additives

The physicochemical properties of the different biocovers employed as odour-reducing additives in this study are presented in Table 2. Biocovers from ash [pH: 13 (C_N_H-A); 12 (CH-A)] and biochar [pH: 9 (B-BC); 8 (RH-BC); 9 (SD-BC)] were alkaline, whereas that from the biomasses [pH: 5 (NS-B); 5 (M-B); 6 (RH-B)] were acidic except for SD-B, which was neutral (pH 7). It is worthy of mention that the pHs of the biochar (RS-BC and SD-BC) were higher than their precursors (RS-B and SD-B). The above results may be a consequence of the thermal treatment processes utilized for the ash and biochar production. Similar reports have been documented in the literature. For instance, Afful et al.^62^ recorded a high pH of 10.49 for coconut fibre and 10.35 for cocoa husk and ascribed the results to the thermal processing of the raw materials. That notwithstanding, the acidic or basic properties of additives influence the microbial growth of organisms and may affect the emission of NH_3_ and H_2_S. This is because at higher pH nitrogen is released as NH_3_ whilst, on the other hand, H_2_S forms sulphides at higher pH thereby reducing the H_2_S release^20^.

**Table 2:** Physicochemical properties of biocovers employed as odour-reducing additives.

Category	Additives	pH	Moisture content (%)	Total volatile solids (%)	Fixed solids (%)	Carbon (%)	C/N ratio
Ash	C_N_H-A	13	3.9	4.2	95.8	2.4	33
	CH-A	12	3.2	15.8	84.2	9.2	125
Biochar	B-BC	9	2.3	92.6	7.4	53.7	98
	RH-BC	8	2.9	60.4	39.6	35	60
	SD-BC	9	5	89.8	10.2	52.1	89
Biomass	NS-B	5	11.1	91.1	8.9	52.8	17
	M-B	5	8.7	90.2	9.8	52.3	13
	RH-B	6	9	84.1	15.9	48.8	103
	SD-B	7	30.8	98.1	1.9	56.9	97

Moisture content for all biocovers was relatively low (< 5% for ash and biochars; within 8.6 to 11.2% for biomass) except for SD-B (30.8%). Less moisture content tends to hinder the growth of organisms, therefore, additives with less moisture content play a significant role in reducing bacterial growth^63^ and consequently, potentially lessening odour-production and release.

Interestingly, the contents of fixed solids were observed to be inversely proportional to the volatile solids. It has been established that, upon increasing temperature for the determination of volatiles, the volatile matter is driven off, burnt away leaving ash, or fixed solids. C_N_H-A and CH-A both had higher fixed solids (respectively, 95.8% and 84.2%) compared to the other biocovers. This is because of the higher temperature they were subjected to, for ashing to take place; thus essentially eliminating the existing volatiles and leaving behind the fixed solids. Also, notice that the fixed solids in the biochars (RH-BC = 39.6%; SD-BC = 10.2%) were higher than that of their corresponding biomasses (RH-B = 15.9%; SD-B = 1.9%). Reasons are similar to those described earlier on in the text.

Carbon content was also generally high for biochars (B-BC = 53.7%; RH-BC = 35%; SD-BC = 52.1%) and biomasses (NS-B = 52.8%; M-B = 52.3%; RH-B = 48.8%; SD-B = 56.9%), and extremely low for ash biocovers (C_N_H-A = 2.4%; CH-A = 9.2%) for reasons attributed to the extent of thermal treatment each biocover precursor material underwent. That notwithstanding, SD-B’s high carbon content may be a consequence of the source of the biomass, *Celtis Mildbraedii*; which is woody^64^. Similar results of the high carbon content of woody biomass have been reported by Sewu et al.^35^.

For C/N ratio, CH-A recorded the highest value of 125 followed by RH-B at 103. Moringa and neem seed powder recorded the lowest C/N ratio of 17 and 13, respectively suggesting higher nitrogen contents relative to carbon contents in these materials. A high C/N ratio has been reported to have an impact on the reduction of odour levels in compost^65^.

### Evaluation of the odour-reduction/removal performances of the applied biocovers

#### Effect of biocovers as additives on H_2_S reduction

**Figure 4:**
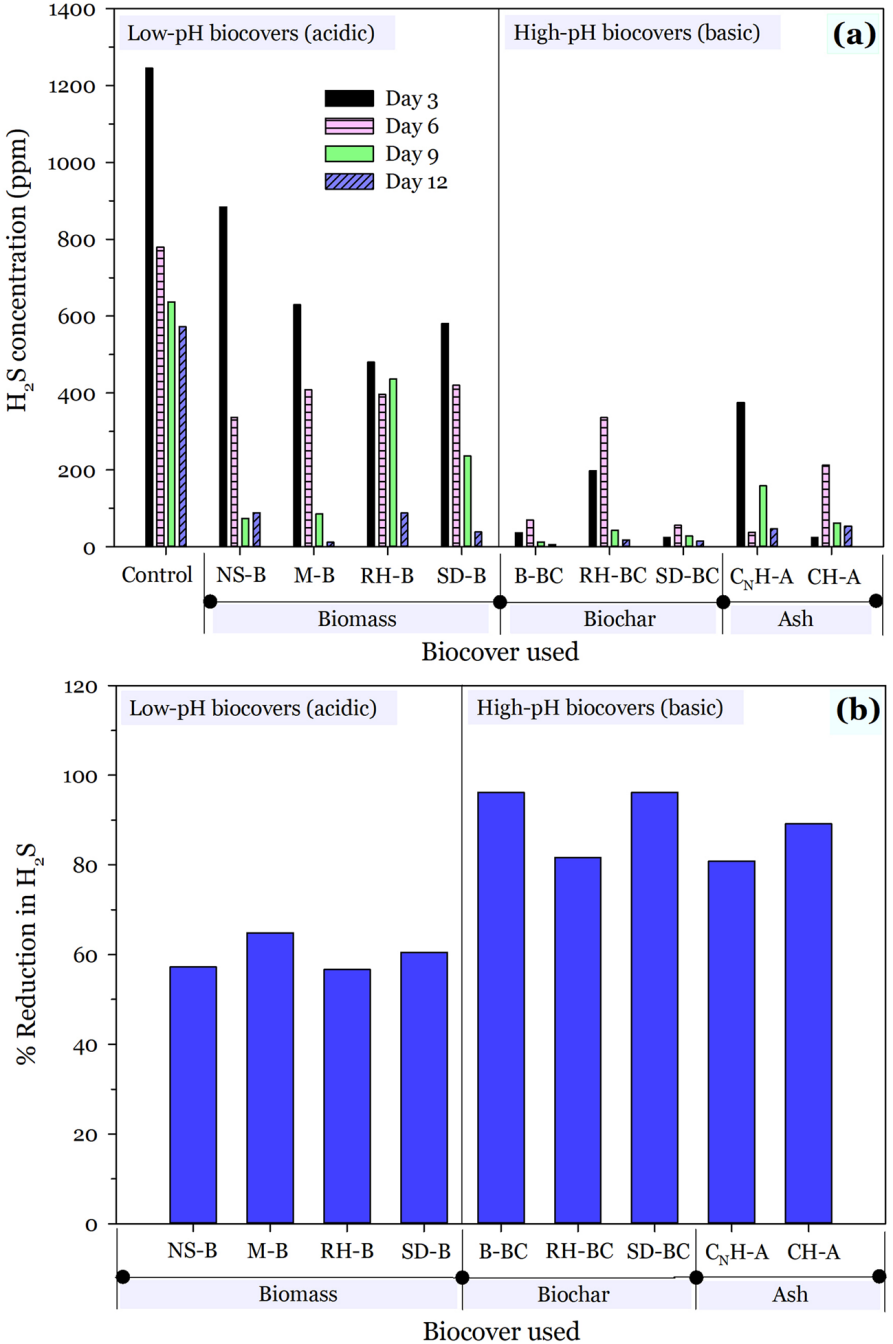
Effect of biocover type and acidity (pH) on mitigation of H_2_S on a (**a**) daily and (**b**) 12 days basis.

The effect of biocovers on the suppression or inhibitions of H_2_S evolution from FS for each studied sampling day over 12 days are shown in Fig. 4a. Results show that apart from the inherent properties of biocovers—its efficacy on H_2_S reduction was time-dependent. Generally, biocovers with pHs in the acidic range required more time to be as effective as biocovers in the basic range. In addition, there was a general decline in H_2_S evolution with FS ageing. H_2_S was released most when FS was freshest at the time of sampling (day 3). This was particularly the case for the control sample and the biomasses (acidic biocovers). The basic biocovers, except for C_N_H-A, rather showed the most release of H_2_S on sampling day 6; with a trend consistently following the order: 6th day > 3rd day > 9th day > 12th day (in terms of concentration of H_2_S released). Furthermore, the application of basic biocovers led to a dramatic decrease in H_2_S evolution on the first sampling day (day 3); which was impressive. For instance, except for C_N_H-A, decreases were over 80% from the control value of 1245 ppm to 37 ppm for B-BC (97%); 198 ppm for RH-BC (84.1%); and 24 ppm for both SD-BC and CH-A (98.1%). This suggests biocovers of basic origin are effective for the rapid attenuation of H_2_S evolution by over 80%.

The results of the overall per cent reduction in H_2_S over the entire study period of 12 days are shown in Fig. 4b. It is evident that, generally, all biocovers performed well (over 55%) in mitigating H_2_S release. However, the extent of H_2_S reduction (%) was greater in the basic biocovers than in the acidic biocovers. Basic materials are known to reduce H_2_S release best since the increase in pH converts H_2_S to sulphides; essentially trapping it within the FS^20^. That notwithstanding, amongst the basic biocovers, biochars were more effective at diminishing H_2_S evolution than ash with a near-complete reduction in H_2_S evolution [biochars: 96.2% (B-BC and SD-BC), 81.6% (RH-BC); ash: 80.9% (C_N_H-A) and 89.1% (CH-A)]. This contrary result of better reduction of H_2_S for biochar than ash, despite the higher pH of ash, may be due to the high surface area, surface functionality and porosity of biochar. Consequently, biochar may adsorb H_2_S thus limiting its release into the atmosphere. The carbon contents may likely be another reason, as the highest performing biocovers [biochar = 96.2% (B-BC and SD-BC); ash = 89.1% (CH-A)] also exhibited the highest carbon contents within the category of biochar (B-BC = 53.7%; SD-BC = 52.1%) and ash (CH-A = 9.2%) for the basic biocovers. Nevertheless, the higher performance of basic biocovers with high carbon content than acidic biocovers with comparable carbon contents for H_2_S reduction suggests that the earlier reasoning about surface area, pH, porosity and surface functionality may better explain the observations than carbon contents. Another plausible explanation may be role of microorganisms in H_2_S biodegradation and/or biotransformation. Microbes such as *Alicyclobacillus* and *Tuberibacillus,* have shown a high positive correlation with H_2_S biodegradation and/or biotransformation^66^ and typically find suitable niches in the applied biocovers. Porous and high surface area biocovers have been shown to provide suitable niches for microbial attachment and growth, and consequent inorganic/organic volatile compounds adsorption^66,67^. Thus, as the diffusion of the evolved H_2_S from the faecal sludge is slowed due to the physical barrier effect of the biocovers, contact with the sulphur-metabolizing bacteria such as *Ochrobactrum*, *Paracoccus*, *Comamonas* and *Pseudomona*^68^ increases thereby yielding better H_2_S attenuation results, especially in the case of biochars, particularly SD-BC. Of the three groups of biocovers used, biochars have the most porosity and surface area hence the observed results.

#### Effect of biocovers on NH_3_ reduction

**Figure 5:**
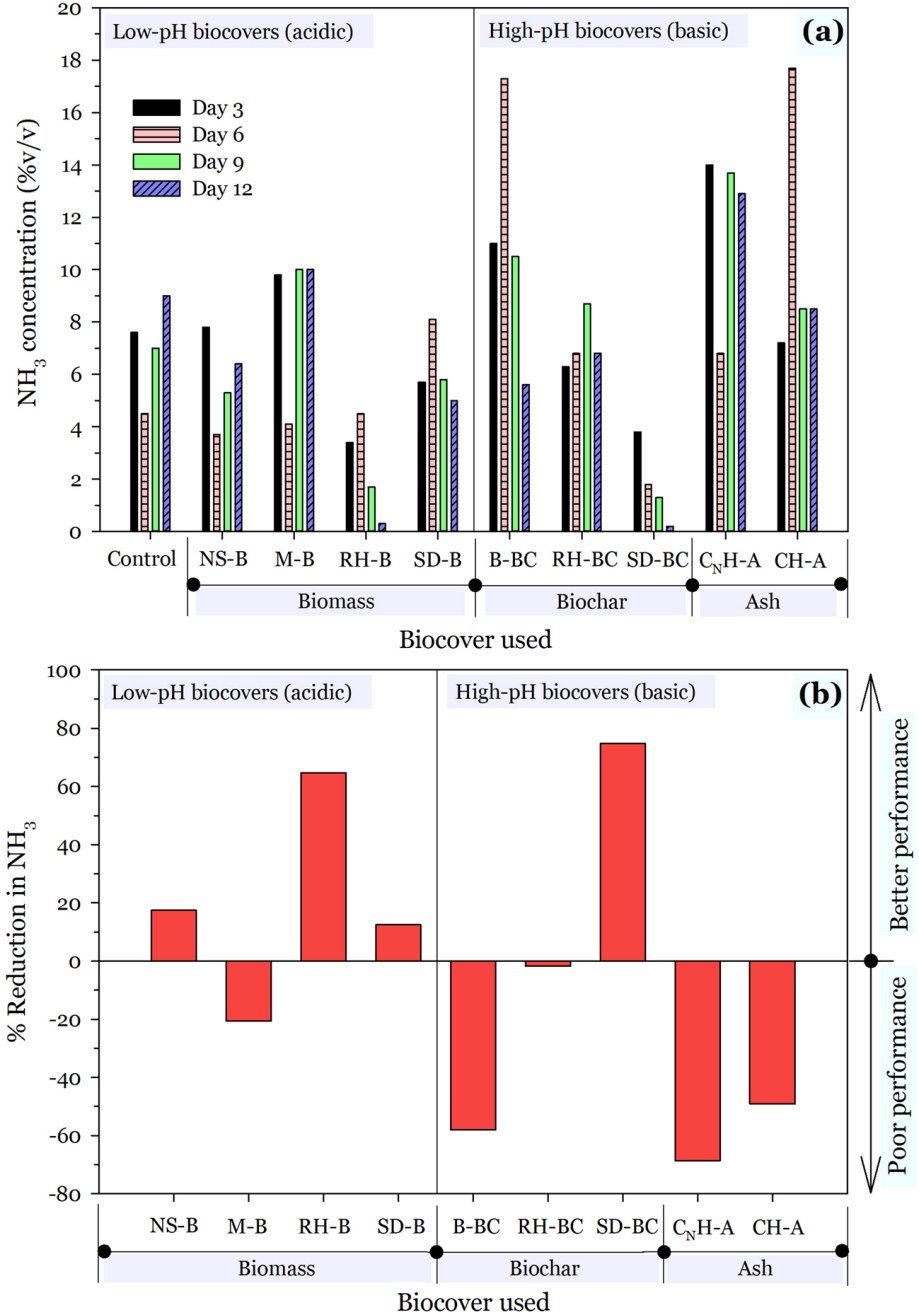
Effect of biocover type and acidity (pH) on mitigation of NH_3_ on a (**a**) daily and (**b**) 12 days basis.

The effect of biocovers on the suppression or inhibitions of NH_3_ evolution from FS for each studied sampling day over the 12 days are shown in Fig. 5a. Generally, there seem not to be a clear trend in NH_3_ suppression with time given the utilized biocovers. No trend consistent with pH values, C/N ratio or carbon content was found in this study. Nevertheless, contrasting results were observed for biomasses and their corresponding biochars. For instance, whilst RH-B exhibited a general gradual decline in NH_3_ evolution with time [6th day (4.5%v/v) < 9th day (1.7%v/v) < 12th day (0.3%v/v)], its biochar (RH-BC) rather displayed an enhancement in NH_3_ evolution with time [3rd day (6.3%v/v) > 6th day (6.8%v/v) > 9th day (8.7%v/v)]. In addition, compared to the control, the decline in NH_3_ evolution was drastic and rapid for all sampling days over the 12 days with the application of SD-BC [3rd day (3.8%v/v); 6th day (1.8%v/v); 9th day (1.3%v/v); 12th day (0.2%v/v)]. A similar observation was also seen for the SD-BC precursor (SD-B) except on day 6; where a heightened NH_3_ evolution rather occurred.

The results of the overall percentage reduction in NH_3_ over the entire study period of 12 days are shown in Fig. 5b. It is evident that majority of the acidic biocovers reduced the emission of NH_3_ much better than the basic materials. In fact, except for SD-BC, all basic biocovers were not just extremely poor at attenuating NH_3_, but rather facilitated its release when compared to the control sample; by 58% (B-BC), 1.8% (RH-BC), 68.7% (C_N_H-A) and 49.1% (CH-A). Conversely, however, except for M-B, the acidic biocovers were good attenuators of NH_3_ evolution. Particularly, RH-B (64.8%) was the most effective amongst the acidic biocovers at attenuating NH_3_ evolution, followed by NS-B (17.4%) and SD-B (12.5%). Interestingly, not only was SD-BC the only effective biocover amongst the basic biocovers, it was also the most effective (74.7%) amongst all the investigated biocovers in this study in attenuating NH_3_ evolution. The plausible reason for this observation was attributed to microbes-related (such as *Rhodanobacter*, *Gemmatimonas*, *Flavisolibacter*, and *Sphingomonas*) biotransformation and/or biodegradation due to the potentially higher surface area and porosity of SD-BC, thus yielding a more conducive environment for microbial attachment and growth^66,69^. The above reasoning was considered the most plausible for the superb performance of SD-BC, because except for the moisture content, its physicochemical properties were comparable to that of B-BC, yet B-BC exhibited a contrary result on NH_3_ performance. According to Atia et al.^20^ the application of the biocovers reduces emissions of NH_3_ and other odorous gases in two ways: (1) physically limiting the emissions of NH_3_ and other gases; (2) creating a biologically active zone on the top of the covers where the emitted NH_3_ and other gases are aerobically decomposed by microorganisms. The effectiveness of different covers or odour-reducing material in mitigating H_2_S and NH_3_ emissions vary and it is also dependent on the quantity of the materials added as a cover. In theory, the effective suppression of odour is influenced by the pH which creates an unfavourable environment for microbial growth, and the physical masking ability of the additives^20,25^.

### Statistical analysis of the effect of biocover type and duration of application on the suppression of odour from FS

The results of the applied statistical analytical tools generated by Microsoft® Excel are shown in Table 3. From the two-way ANOVA, the computed *F* values for the source of the variations [biocover source = 3.78 (H_2_S), 3.39 (NH_3_); duration of application = 5.04 (H_2_S), 4.76 (NH_3_)] were all, greater than that of the *F crit* values (2.36 for biocover source; 3.01 for the duration of application). In addition, the *P-values* were lower than the level of significance at 0.05. From the aforementioned results, it was evident that the effects of the independent variables (biocover source and duration of application) on the suppression of both H_2_S and NH_3_ evolution were statistically significant. These deductions were made based on two criteria: *F* value and the *P-value*. Values of *F* greater than reference *F crit*, and *P-values* lesser than the set level of significance at 0.05 (95% confidence limit) are indicative of a significant contribution to the variation by the group under investigation. Also, it was evident from the one-way ANOVA that variations in the means between the biocover type (biomass, biochar, ash) were statistically significant for H_2_S [*F* (26.34) > *F crit* (5.148); *P-value* (0.0011) < 0.05] and insignificant for NH_3_ [*F* (1.956) < *F crit* (5.148); *P-value* (0.223) > 0.05]. Consequently, for the H_2_S, Tuker-Kramer multiple comparison test was used to evaluate which pair or combination of biocover type was the source of the variations for suppression of H_2_S evolution from FS. Clearly, the significant variations arose with biomass (low pH) and biochar/ash (higher pH) pairs suggesting that for effective H_2_S suppression, the pH of the biocovers is essential.

**Table 3:** Results of the applied statistical analysis tools for the interpretation of odour suppression data (%).

Source of variation	df	*F* crit	H_2_S suppression		NH_3_ suppression	
			*F*	*P*-value	*F*	*P*-value
**Two-way ANOVA without replication**						
Biocover source	8	2.36	3.78 (S)	0.0058	3.39 (S)	0.0097
Duration of application	3	3.01	5.04 (S)	0.0075	4.76 (S)	0.0096
**One-way ANOVA**						
Between the groups of biocover type	2	5.143	26.34 (S)	0.0011	1.956 (NS)	0.223

Tukey–Kramer multiple comparison	Biocover type					
	H_2_S suppression (C.R. = 4.339)			NH_3_ suppression (C.R. = n.a.)		
	Biomass	Biochar	Ash	Biomass	Biochar	Ash
**Tukey–Kramer multiple comparison test results on the effect of biocover type on overall odour suppression efficiency**						
Biomass	X	9.68 (S)	6.82 (S)	–	–	–
Biochar	–	X	1.63 (NS)	–	–	–
Ash	–	–	X	–	–	–

**NB:** Biocover source = (NS-B, M-B, RH-B, SD-B, B-BC, RH-BC, SD-BC, C_N_H-A, CH-A); Biocover type = (biomass, biochar, ash); duration of experiment, days = (3, 6, 9, 12); C.R. = critical range; *df* = degree of freedom; *F* = determined from experimental data using the *F-*test*; F crit* = *F* statistic obtained from the *F*-distribution; *P-value* = probability value; S = significant (absolute difference between mean odour suppression efficiencies is significant); NS = not significant (absolute difference between mean odour suppression efficiencies is not significant); n.a. = not applicable.

### Economic analysis and feasibility studies

Determining the cost associated with using particular technologies is essential to the scale-up and ultimate success of the technology in a real application. Consequently, cost analysis was determined based on (1) accessibility of the raw materials for the biocover production; (2) processing/production of the biocovers; (3) performance of biocovers on H_2_S and NH_3_ evolution; and (4) longevity of biocover. On the raw material accessibility, all materials were obtained as waste, thus the only cost likely to be incurred will be truck delivery and tipping fee, which hypothetically will be the same for all raw materials when factors such as proximity to the biocover production site and end-use (application) site remain the same. For the processing, however, the cost will follow the trend: biomass > biochar > ash. The main operation in the biomass processing was drying or used as-is. For biochar and ash, however, temperatures of 400 °C and 700 °C were used, respectively. Using a performance criterion of above 50%, it was apparent that from Figs. 4b and 5b, only RH-B (biomass: acidic) and SD-BC (biochar: basic) both exhibited H_2_S (56.6% and 96.2%, respectively) and NH_3_ (64.8% and 74.7%, respectively) attenuation above the 50% threshold, for potential adoption in real application. However, for environmental considerations, RH-B may itself, decompose and release greenhouse gases (GHG) into the atmosphere owing to the abundance of labile carbon fractions in the biomass^70^. Conversely, SD-BC (biochar) which has a long carbon half-life contains aromatized carbons, which are unavailable for microbial consumption and can persist, therefore, for longer times (years)^71^ thus increasing its overall utility, lessening application frequency and improving climatic conditions via carbon sequestration. Moreover, the pyrolysis process is considered a closed system and can be self-sustaining, where the other pyrolysis products (pyrolytic gases and bio-oil) are reutilized as fuel for running the process^72^, thus leading to drastic cost reduction. From the above analysis, employing SD-BC as biocover for H_2_S and NH_3_ mitigation is feasible on a cost and removal efficiency basis.

## Conclusions

This study assessed the potential of employing plant waste materials, biochar and ash as biocovers to attenuate foul odour evolution from faecal sludge in a laboratory setting. Results showed that odour levels, assessed with H_2_S and NH_3_ as indicators, in the two public latrines were above the perceptible threshold of 0.005 ppm (H_2_S) and 0.05 ppm (NH_3_) for humans and peaked in the mornings and evenings—correlated with patronage times. Comparing the odour-causing substances, H_2_S and NH_3,_ only the former was above the threshold of unbearableness/annoyance of 0.05 ppm (H_2_S) and 30 ppm (NH_3_) to humans in the toilets investigated. Characterization studies showed that the biomasses were acidic whereas the biochars and ashes were basic. Odour-suppression results showed that generally, high pH biocovers were more effective at suppressing H_2_S evolution from FS. The effect of pH on the suppression of NH_3_ was determined to be statistically insignificant at 95% confidence limit. The per cent H_2_S and NH_3_ reduction values were the highest for biocover from sawdust biochar; 96.2% and 74.7%, respectively. These results suggest that waste and readily available resources such as sawdust biomass, when converted to biochar, can serve as an effective tool to attenuate odour evolution from fresh faecal sludge in dry sanitation public toilets.

## Supplementary Information

Supplementary Figure S1.

## Data Availability

Authors declare that data can be available upon request from the corresponding author.
