# Supplementary material for: Inhibition of ammonia and hydrogen sulphide as faecal sludge odour control in dry sanitation toilet facilities using plant waste materials
Source: Sci Rep. 2021 Sep 7;11:17803. doi: 10.1038/s41598-021-97016-w (PMC8423729; doi:10.1038/s41598-021-97016-w)
Supplement: Supplementary file 1 — Supplementary Figure S1. [file 41598_2021_97016_MOESM1_ESM.docx]

**Supplementary Information**

**Inhibition of ammonia and hydrogen sulphide using plant waste materials for faecal sludge odour control in dry sanitation toilet facilities**

Bernice Mawumenyo Senanu ^a^, Patrick Boakye ^b^*, Sampson Oduro-Kwarteng ^a^, Divine Damertey Sewu ^c, d^, Esi Awuah ^a^, Peter Appiah Obeng ^e^, and Kobina Afful ^a^

*^a^ Department of Civil Engineering, Kwame Nkrumah University of Science & Technology, UPO, PMB, Kumasi, Ghana.*

*^b^ Department of Chemical Engineering, Kwame Nkrumah University of Science & Technology, UPO, PMB, Kumasi, Ghana.*

*^c^Life Green Technology Co. Ltd., 875 Yuseong-daero, Yuseong-gu, Daejeon 34158, Republic of Korea*

^d^*Department of Chemical and Biological Engineering, Hanbat National University, 125 Dongseo-daero, Yuseong-gu, Daejeon 34158, Republic of Korea*

^e^*Department of Water and Sanitation, University of Cape Coast, Cape Coast, Ghana*

------------------------------------------------------------------------------------------------

** Corresponding authors. Tel.: +233 (0) 244109589.*

E-mail address*:* [*patrickboakye@knust.edu.gh*](mailto:patrickboakye@knust.edu.gh)


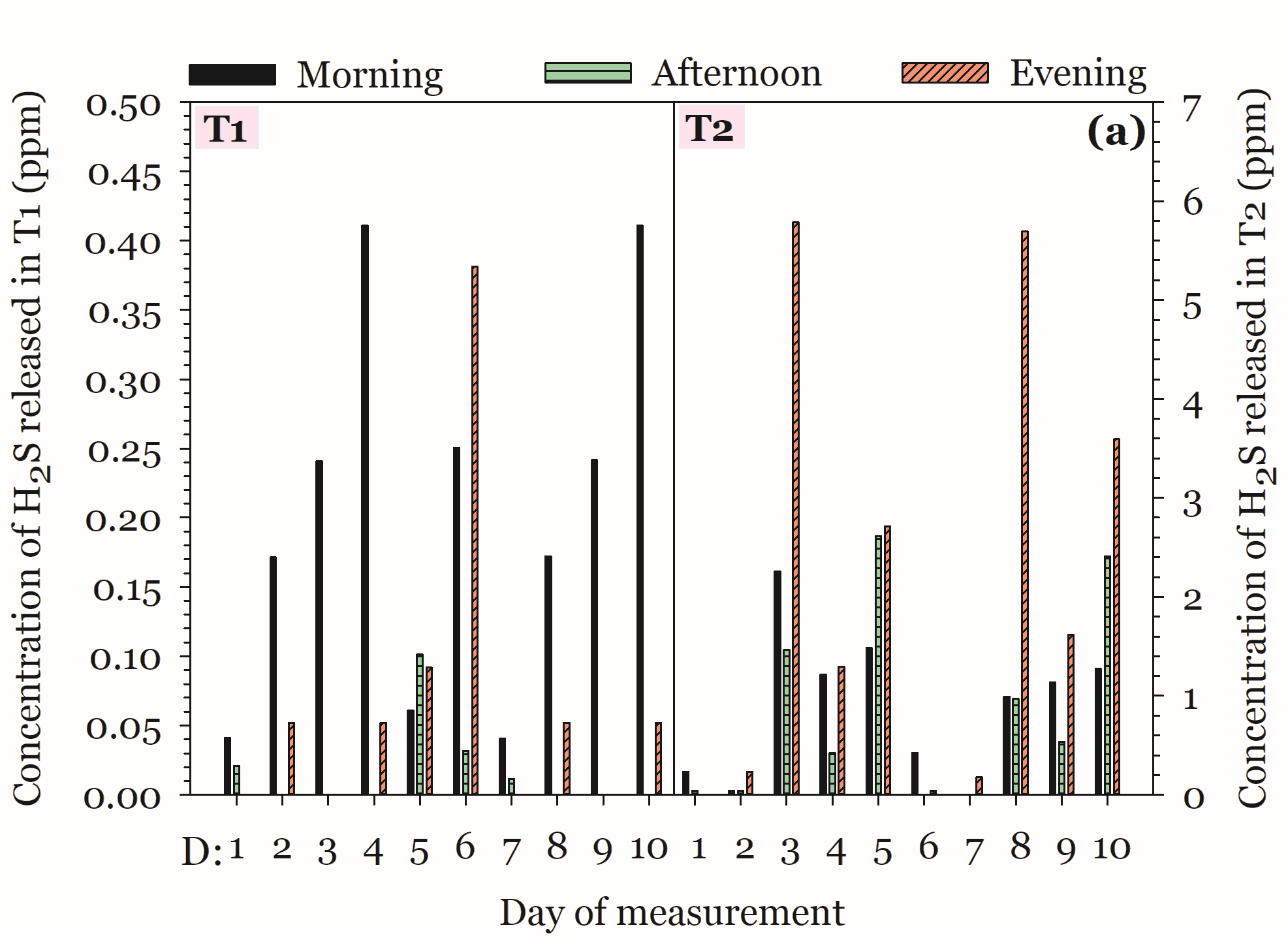

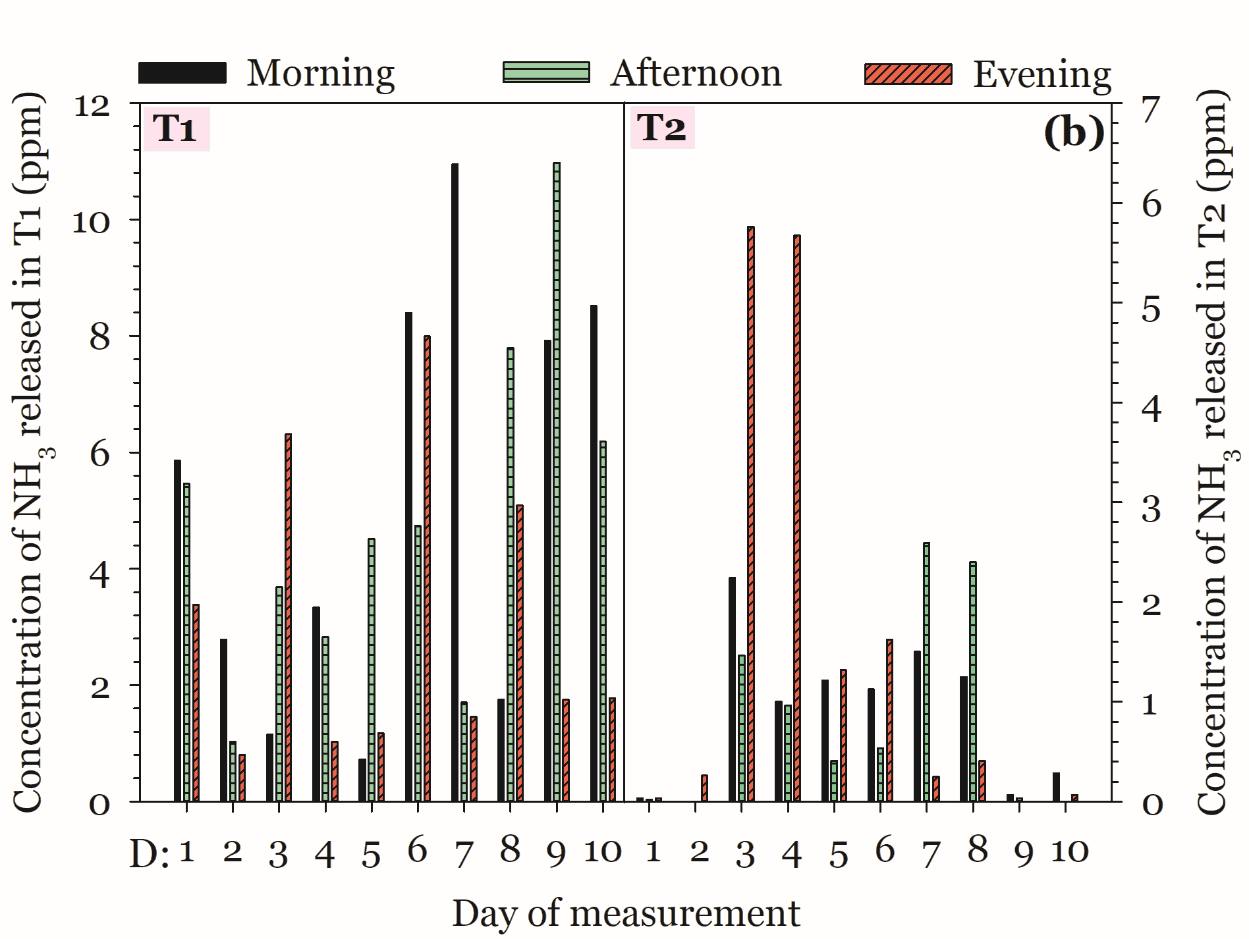


**Fig. S1.** The concentration of (a) H_2_S and (b) NH_3_ released from T1 and T2 over 10 days at different sampling times (mornings, afternoons and evenings) within the day.
